# New Paradigm for a Targeted Cancer Therapeutic Approach: A Short Review on Potential Synergy of Gold Nanoparticles and Cold Atmospheric Plasma

**DOI:** 10.3390/biomedicines5030038

**Published:** 2017-07-01

**Authors:** Sajesan Aryal, Gunjan Bisht

**Affiliations:** 1 Department of Biotechnology, School of Science, Kathmandu University, Dhulikhel 45200, Nepal; asajesan04@gmail.com; 2 Department of Chemical Science and Engineering, Kathmandu University, Dhulikhel 45200, Nepal

**Keywords:** gold nanoparticles, atmospheric cold plasma, tumor-specific targeted therapy

## Abstract

Application of Gold nanoparticles and Cold Atmospheric plasma as a targeted therapeutic adjunct has been widely investigated separately in cancer therapy. Gold nanoparticles, with their biocompatibility, lower cytotoxicity and superior efficacy, are becoming substantially more significant in modern cancer therapy. Likewise, cold atmospheric plasma, with rich reactive species including reactive oxygen species (ROS) and reactive nitrogen species (RNS), is being explored to selectively target and kill cancer cells, making them a promising anticancer agent. Recent scientific studies have shown that there is a potential synergy between these two aspects. Induction of apoptosis/necrosis due to oxidative stress may be a probable mechanism of their cytotoxic effect. The synergetic effect of the two therapeutic approaches could be tantamount to maximized targeted efficacy on the treatment of diseases like cancer.

## 1. Introduction

Cancer, a lethal disease, is driven by huge genetic alterations in normal body cells accumulating attributes like over-proliferation, evasion of apoptosis, sustained angiogenesis, tissue invasion and metastasis [1]. Still, the employed cancer therapies include surgery, chemotherapy, and radiation therapy. Although these orthodox therapies have been practiced for decades, they exhibit adversary effects on cancer patients, as they lack selectivity towards tumor cells, and have detrimental effects on healthy cells [2]. Over the past several years, a new realm of cancer treatment—targeted cancer therapies—has come to the forefront [3]. Research findings from tumor-targeted cancer therapeutic approaches could be a panacea in cancer treatment.

In particular, areas like nanomedicine and plasma medicine are getting attention for the ablation of chronic diseases like cancer. Nanomedicine, due to its advanced imaging applications and wide range of engineering capabilities, holds potential therapeutic applications, along with its capacity for early detection of cancer. Furthermore, nanoparticles have additional advantages of active/passive targeting, bioavailability and biocompatibility over orthodox therapies. Recent advancement has led to the development of bioconjugated functional nanoparticles whereby certain peptides, proteins, nucleic acids and small molecule ligands are covalently attached to them [4,5]. Similarly, for drug delivery, structures like nanospheres (where the drug is dispersed throughout the material) or nano Capsules (where the drug is confined to an aqueous or oily cavity surrounded by polymeric membrane) can be used [6]. Nanoparticle-based toxicity includes ROS generation and oxidative stress, inflammation, DNA damage and inhibition of cell division [7].

Plasma medicine, due to its selectivity mechanisms and efficacy for sterilization of surfaces, has been proposed for selectively targeting cancer cells. Though early research shows promising results, downstream mechanisms have not yet been fully understood. Some of the proposed mechanisms includes oxidative stress and ROS generation, activation of *p53* gene, activation of p21 CKSinhibitor and cell cycle arrest [8].

Cold atmospheric plasma (CAP) can be used as a novel light source. CAP is an ionized gas where the ion temperature is close to room temperature. It contains electrons, charged particles, radicals, various excited molecules, UV photons, and transient electrical fields. CAP spectra cover the range of 250–800 nm. These various compositional elements have the potential to either enhance cellular activity or disrupt and destroy cells based on different cellular structures and different cell resistance to this treatment. For example, a combined treatment may easily improve cancer cell permeability, since cancer cell membranes are more vulnerable than healthy cells, resulting in a greater uptake of nanoparticles. In addition, the effect of gold nanoparticles on the depth of photodynamic treatment with significant potential for their application in oncology has already been studied and established. Using gold nanoparticles and cold atmospheric plasma has shown some very promising results rather than their single use. The results in in-vitro conditions have clearly shown the synergy between the two. In this short review, we would like to explore both of them as a therapeutic modality, and examine how their synergistic approach has been showing promising results in vitro.

## 2. Gold Nanoparticles as Therapeutic Modality

It is apparent that nanotechnology has been showing the huge potential impact on biological and biomedical applications [9]. It is a new, multi-disciplinary scientific research field encompassing physics, chemistry, biology, engineering, medicine and much more. In recent years, significant research probes have been inclined towards developing and promoting nanotechnology for early detection, molecular imaging, accurate diagnosis, and therapeutic models for many diseases including cancer [4,10]. Nanotechnology basically emphasizes on designing, synthesizing and manipulating physical, chemical and biological properties of materials at the nanoscale (1–100 nm); which ultimately integrates well into the biological system, just as viruses or bio-molecules do [11,12]. These small-sized nanoparticles confer substantial variation in their optical, electronic, magnetic and structural properties distinct from their bulk materials, which can further be exploited for various medical applications [5,13]; therefore, these nanoparticles (NPs) are emerging as novel and improved therapeutic and imaging agents in cancer therapy [6]. The arena of nanotechnology dealing with disease diagnosis, monitoring and treatment has been referred as “nanomedicine” by the National Institutes of Health in the USA [14]. The fundamental aim of nanoparticle (NP)-mediated cancer therapy is to target and monitor therapeutic activity to the tumor while sparing the healthy and normal tissue [11].

**Figure 1:**
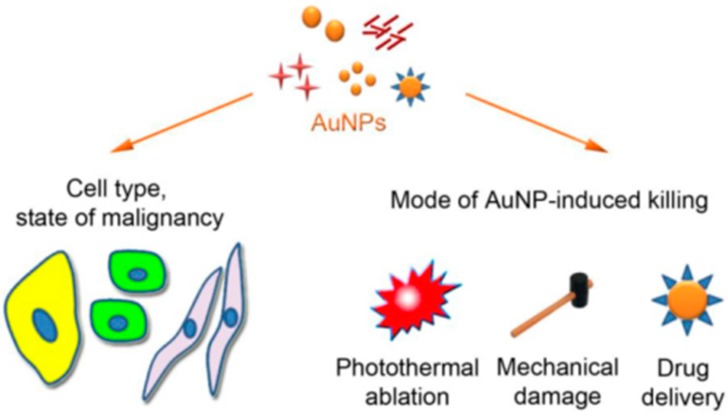
Depicting the cytotoxic impact of gold nanoparticles on tumor cells. This picture represents three of the major mechanism of cytotoxicity conferred by gold nanoparticles-photothermal damage, mechanical damage and drug delivery. Gold nanoparticles (GNPs) are referred as AuNPs here.

Among substantial nano-materials being studied for nanomedicine applications, gold nanoparticles (GNPs) are being explored as a paradigm because of (a) their shape, size and surface chemistry, which can be easily controlled and modified; (b) their biocompatibility and lower cytotoxicity [15]; and (c) their capacity to be used as tumor specific drug carrier agents, imaging agents, radiosensitizers, and antiangiogenic agents [16]. The therapeutic value of GNPs are based on their various properties; enhanced permeability and retention (EPR) being one which facilitates their infiltration inside tumors [17]. Multiple in vitro studies show that GNPs confer their cytotoxicity in cells by induction of oxidative stress, mechanical damage, photothermal ablation and drug delivery [16,17,18]. Gold nanoparticles, due to their surface Plasmon resonance effect, can absorb strongly in visible and near infrared regions, and are very well suited for applications in cancer phototherapy [19,20]. As depicted in Figure 1, GNPs can destroy cancer cells by photothermal ablation, mechanical damage or by drugs used in cancer treatment.

However, the key challenges when using gold nanoparticles in cancer therapy are biodistribution, and possible cytotoxicity. Proper characterization of nanomaterials and a good animal model with substantial sample size and good statistical inference are necessary for the understanding of the biodistribution of the particle [21]. Furthermore, knowledge about their cytotoxicity and health impact should be robust before using it in real clinical settings. One of the key features for understanding it may be the study of its impact on physiological body fluids [22].

## 3. Cold Atmospheric Plasma as Therapeutic Modality

**Figure 2:**
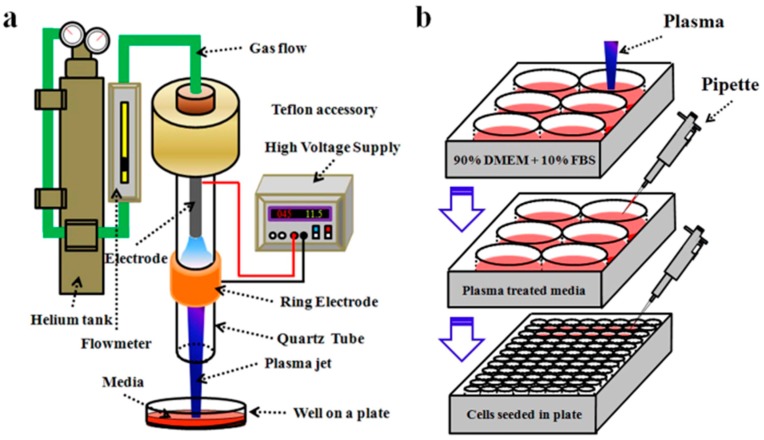
Schematic diagram showing the two methods of plasma treatment. (**a**) Direct treatment on cells with media and also depicts the general plasma generation process. Helium gas acts as a carrier gas for continuous generation of plasma (green line). Plasma (blue line) is generated in between the two electrodes by the application of high voltage. The generated plasma jet can directly be exposed to the cell culture vessels; (**b**) Exposure of cells into plasma treated media. (Dulbecco’s Modified Eagles Medium (DMEM) is cell culture medium used in conjugation with Fetal Bovine Serum (FBS)).

The notion of the realm of Plasma Medicine is yet another recent, largely unexplored, but potentially beneficent scientific field that has gained critically acclaimed interest for cancer researchers in the past several years [23]. Plasma, considered the fourth state of matter, is what makes up the most part of the visible universe [8,23]. The idea of plasma generation is fairly simple; when gas is heated further electrons are completely separated from the nucleus component of atoms to form a plasma [8]. Briefly, classifying plasma can be subdivided into two types: thermal plasma and non-thermal/cold plasma. Thermal plasma consists of knocked-off electrons and heavy particles at thermal equilibrium. In contrast, non-thermal or cold plasma has a non-equilibrium system where electrons have much higher temperature than heavy particles [24]. Recent advances in plasma technology have laid the foundation for the generation of cold plasma with a temperature less than 40 °C, making it ideal for interaction with living tissue [8], especially in therapeutic and medical applications [25]. Figure 2 represents the schematic diagram of the generation of plasma jets in the laboratory and their usage in cell lines. The generation of plasma jets in lab requires energy (thermal, electrical or light). Usually, the discharge necessary to produce it is induced electrically [26]. Two types of plasma devices—the plasma jet and dielectric barrier discharge—have been widely used in the plasma medicine field. Plasma is generated in between two electrodes (anode and cathode). Either anode or cathode is covered by dielectric material such as quartz as shown in the Figure 2. Also plasma jet devices require a carrying gas—either helium or argon—the continuous flow of which helps to produce the plasma jet [27].

Though the elucidation of the exact mechanism of CAP response on tumor cells still remains enigmatic, several mechanisms have been proposed, including apoptosis via reactive oxygen species, reactive nitrogen species and cell cycle disruption [8,23]. CAP uniquely confer a rich environment of reactive oxygen species (ROS), reactive nitrogen species (RNS), electrons, charged particles, photons, and electric fields [8,28]. Still the controversy with respect to plasma-cell interaction has not yet been well comprehended [29]. Some researchers explain that charged particles in plasma composition play a vital role while others believe neutral particles as significant effectors. At the same time, CAP are highly selective, as molecules like oxygen promote a plasma-killing effect, and molecules like nitrogen produce a plasma-healing effect [23]. The variety of different plasma effects can be explained by the complexity of their chemical composition, including neutral molecules, atoms, nitric oxide (NO), hydroxyl radicals (OH), ozone molecules (O_3_), oxygen atoms (O), and the variations in their generation mechanism [8,30]. However, CAP produced by generating a unique chemical environment similar to endogenous RNS/ROS can intervene in abounding cell signaling pathways. With the aid of these potentially selective, highly reactive species, targeting tumor cells selectively without conferring any detrimental effects on surrounding normal tissue can be achieved [8]. The major challenges of using cold plasma as a therapeutical approach is the lack of detailed clinical tests about its interaction with the body, and its long-term negative effects. Successful clinical trials should be carried out for reliable therapeutic effects.

## 4. Biocompatibility

Gold nanoparticles are generally less cytotoxic, and have potential use in nanomedicine [31]. The generation of ROS is an important toxicity-confering mechanism of GNPs, which may disturb the equilibrium between oxidant and anti oxidant cellular processes. Also, DNA damage in normal cells is less sensitive than in cancer cells upon GNP exposure [32]. However, recent studies have suggested that the size-dependent cytotoxicity of gold nanoparticles, decreasing size of NPs correlated with deeper penetration inside cells and more toxic effects [16]. Similarly a detailed review by Waltmann and Woedtke escribes cold plasma as being very useful for medical purposes like wound healing and tissue regeneration, highlighting its biocompatibility and also its anti cancer effects [33]. Reactive oxygen and nitrogen species generated after plasma exposure plays a determining role in biological responses. The biological plasma effect also depends upon the change of liquid environment in cells. The higher susceptibility of cancer cells towards these effects might be attributed to their higher metabolism rate, alteration in pH and abnormal cellular functions.

## 5. Selectivity

Selectivity is the foremost consideration to be made for the treatment of disease like cancer, and the study of the working mechanism of both Gold nanoparticles (GNPs) and Cold Atmospheric Plasma (CAP) reveals their potential selectivity towards cancer cells. The major downstream effect inside cells after their exposure would be the production of ROS/RNS [34]. Oxidative stress inside cells is either attributed to cellular processes like mitochondrial respiration and immune cell activation, or to cell-nanoparticle interactions [7]. Furthermore, in general, ROS production is higher inside cancer cells than in normal cells [34,35,36]. One possible cause might be the higher metabolism rate inside cancer cells compared to normal cells [36]. This could provide the potential selectivity of ROS/RNS for killing cancer cells.

A comprehensive review published by Kong et al. highlights how plasma and nanoparticles interact with cells, and why plasma nanotechnology seems so promising in medicine [34]. A recent study by Zhu et al. demonstrated that drug-loaded nanoparticles and CAP synergistically increased inhibition of breast cancer cell growth in comparison to their separate treatment. They also found downregulation of metastasis-related genes upon CAP treatment, along with facilitated uptake of drug-loaded nanoparticles [30].

## 6. Gold Nanoparticles (GNPs) Meets Cold Atmospheric Plasma (CAP)

CAP generated by specific devices in labs has just recently been emerging as an anticancer therapeutic agent, as shown by substantial research studies [37]. In a parallel way, GNPs are the most studied among all metallic nanoparticles, and they confer potential anticancer effects due to their photophysical and surface chemistry [38]. Cold Atmospheric Plasma being the combination of light energy, reactive chemical species and charged particles, there is enough space to define a synergy between CAP and GNPs [39]. The overall synergy by integration of GNPs and CAP as a therapeutic approach provides a promising tool for developing newer cancer therapeutic strategies, which go beyond simple combination, and can potentially shed new light on cancer therapy [28].

## 7. Synergy of GNPs and CAP: A Promising Anticancer Approach

The leadoff result in this arena came in a study by Kim et al., where they used antibody-conjugated 30 nm gold nanoparticles to show a fivefold increase in G361 human melanoma skin cancer cell death over the case with plasma alone. The same study also concluded that even in low plasma doses, CAP can stimulate GNPs located inside cells to cause cell death [40] Another study also indicated a potential synergy between GNPs and CAP in cancer therapy. They showed that treatment with CAP along with GNPs significantly induced U87 glioblastoma human brain cancer cell death, withup to a 30% overall increase compared to the control group with plasma treatment but no GNPs [39]. Furthermore, gold nanoparticles were endocytosed with the significantly accelerated rate in U87 cell membrane when treated with plasma, while no significant difference in gold nanoparticle penetration in normal cells was observed [28,41]. It was concluded that nanoparticles coupled with cold plasma would decrease cell viability, allowing CAP to be more efficient [41]. An additional recent result indicated that cell death was increased significantly by the treatment of cold plasma in the presence of gold nanoparticles [42]. Actually, they showed HTC 116 human colorectal cancer cells when treated with 55nm GNPs with 375 ppm concentration with plasma treatment for 180 s increased apoptotic cell death. Their result also correlates well with the theory that significant increase in accumulation of intracellular ROS inside cells treated with GNPs coupled with CAP leads to apoptotic cell death [42]. A very recent study by Kaushik et al. showed that the co-treatment of 100 nm PEG-coated gold nanoparticle and cold plasma (for 150 s) was able to significantly decrease proliferation in Glioblastoma multiforme T98G and lung adenocarcinoma A549 cancer cells by abrogating the PI3K/ATK signallingpathway. The same study also showed the reversal of epithelial-mesenchymal transition (EMT) as confirmed by both in vitro as well as in vivo study on tumor cells following treatment with CAP and GNP [43]. From these studies, one can summarize that combining the pros of GNPs and CAP together could be a boon in many aspects, like enhancing the effects of plasma, even at low doses, and promoting GNPs’ effect on and uptake by the cell. In addition, directing therapeutic approaches this way could lead to an overall decrease in cytotoxicity, with increased targeted efficacy [28]. Moreover, a recent study has pointed out the combination of cold atmospheric plasma and iron nanoparticles in decreasing the viability of cancer cells [44].

## 8. Conclusions

The substantial bodyof research over past decades on the effect of gold nanoparticles and atmospheric cold plasma on tumor response separately has increased our knowledge regarding their key roles in ameliorating cancer progression. From the direct delivery method to the development of specific targeted coupled GNPs and CAP, cancer researchers should focus on this arena. Through a better comprehension of the systemic behavior and responses of GNP and CAP-based therapeutics, we might find a new light to counter a multifactorial lethal disorder like cancer. Although a lot of challenges like development of an effective delivery system, optimization of an optimal safe concentration of both GNPs and CAP, and enhancement of the effective coupling of GNPs and CAP to ultimately increase the efficacy of therapy still remain, it is likely that abundant research probes and substantial elucidation of their working mechanisms will help overcome the problems of realizing the full therapeutic potential of GNPs and CAP in upcoming days. To sum up, we might possibly see an effective synergy between the implication of gold nanoparticles and cold atmospheric plasma to provide a greater impetus for the treatment of cancer as a manageable disease, even if not a complete cure.

## 9. Future Standing

This short review describes the application of GNPs and CAP in the therapeutic area, as they are able to selectively induce apoptotic cell death in cancerous cells through oxidative stress. Their coupling can promote the existing oxidative stress they confer separately in the cells when administered.

Future studies should focus on optimization of an optimal safe concentration of coupled GNPs and CAP, which causes minimal adversary effects on normal cells while targets only cancerous cells. Furthermore, exploration of the physical and chemical surface properties of GNPs so that uniquely generated plasma can be adsorbed and be directed in abnormal tissues can be imagined [28]. Localization of plasma can be achieved by their coupling. Substantial studies need to be made to effectively couple GNPs and CAP to increase therapeutic value and ultimately establish a completely new era of plasma nano-oncology.
